# Using visual imagery to manipulate recognition memory for faces whose appearance has changed

**DOI:** 10.1186/s41235-025-00671-0

**Published:** 2025-09-30

**Authors:** Michelle M. Ramey, Darya L. Zabelina

**Affiliations:** Department of Psychological Science, University of Arkansas, Fayetteville, AR 72701 USA

**Keywords:** Eyewitness memory, Episodic memory, Recognition, Visual imagery, Face recognition

## Abstract

Real-world recognition requires our memory system to accommodate perceptual changes that occur after encoding; for example, eyewitnesses must recognize perpetrators across changes in appearance. However, it is not clear how this *flexible recognition* ability can be improved: Standard encoding strategies not only tend to be ineffective, but can in fact be detrimental for recognizing people across appearance changes. Given the effectiveness of visual imagery in creating and modifying memory representations, we examined whether counterfactual visual imagery could be used to manipulate flexible recognition by simulating an increase in encoding–retrieval similarity. Across two experiments, participants (*n* = 317) encoded faces with neutral expressions and were cued to imagine the faces with either happy or angry expressions. During later retrieval, participants saw lineups of old and new faces with either happy or angry expressions, and selected the old face and provided recognition confidence. Old/new recognition discriminability and confidence were higher when a face’s expression at retrieval matched the expression that it was imagined in during encoding (i.e., congruent imagery); interestingly, however, there was Bayesian evidence for no benefit of imagery congruence for face-choice accuracy. Moreover, congruent imagery improved recognition for old arrays irrespective of whether participants correctly selected the old face, suggesting that the imagery manipulation influenced a diffuse sense of recognition without influencing the ability to attribute that sense of recognition to a specific stimulus. Together, these findings indicate that visual imagery can directionally manipulate recognition for changed faces and produces a novel dissociation between old/new recognition and forced-choice accuracy.

**Supplementary Information:**

The online version contains supplementary material available at 10.1186/s41235-025-00671-0.

## Introduction

After we meet someone, we expect to be able to recognize them the next time we see them—even though nearly everything about the person’s appearance (e.g., their expression, hair, and clothing) may differ from when we met them. Similarly, when we return to our hometown for the first time in a decade, we expect to recognize it despite the changes it has undergone. The ability to recognize an entity across perceptual changes, which we term *flexible recognition*, is required for nearly every real-world use of memory. In fact, the ability to flexibly recognize changed entities is critical for even the most consequential uses of memory, such as eyewitness memory and missing persons identification (Juncu et al., 2020; Lampinen et al., 2012; Moore et al., 2021, 2024; Or et al., 2023). Despite the importance of flexible recognition, however, we are surprisingly poor at it (Young & Burton, 2017, 2018)—and it is unclear how this ability could be improved.

A large body of work, particularly within eyewitness memory, has clearly demonstrated that changing perceptual aspects of stimuli between encoding and retrieval dramatically impairs recognition relative to when no changes are made. For example, when the lighting or stimulus angle changes, or when a person changes their clothes, facial accessories, or even facial expression, recognition performance decreases (Brooks et al., 2002; Bruce, 1982; Colloff et al., 2021; Lim et al., 2022; O’Toole et al., 1998; Or et al., 2023; Shapiro & Penrod, 1986; Terry, 1994; Vakil et al., 2019). Interestingly, warning participants that the stimuli will change between study and test not only does not improve performance, but often backfires and impairs performance (Charman & Wells, 2007; Suresh et al., 2025). A perhaps even more puzzling aspect of flexible recognition is that standard manipulations that improve recognition performance for unchanged stimuli often do not improve recognition for changed stimuli. In fact, some manipulations that consistently improve standard recognition memory instead worsen recognition memory for changed stimuli (Read et al., 1990; Vakil et al., 2019). For example, in a study particularly relevant to missing persons identification, Read et al. (1990) examined participants’ recognition for faces across age changes. As expected, increasing encoding duration improved recognition for minimally changed faces; however, increasing encoding duration *worsened* recognition for highly changed faces relative to shorter durations. Similar findings have also been observed with respect to facial expression changes across study and test (Vakil et al., 2019). To explain these paradoxical effects in which standard encoding strategies lead to poorer flexible recognition, it has been proposed that standard encoding strategies typically produce a more rigid representation that over-weights specific sensory details rather than an abstracted representation that allows for more flexible recognition across changes (Read et al., 1990).

If standard strategies for improving recognition are not particularly useful—or are even detrimental—for flexible recognition, how can flexible recognition be improved? One particularly promising avenue is increasing variability of stimuli during encoding: When participants are shown a large variety of pictures of a person during study—rather than an equivalent number of repeated pictures—later recognition for a novel picture of that person is typically improved (Honig et al., 2022; Juncu et al., 2020; Murphy et al., 2015; Ritchie & Burton, 2017). This has been interpreted to reflect that more flexible memory representations are formed when there is high variability of exemplars during encoding. However, Honig et al. (2022) recently demonstrated that the benefits of encoding variability are driven by an increased probability of studying an image that ends up being highly similar to the tested image (e.g., the person to be recognized happens to be shown from the same angle at study and test). Therefore, rather than improving flexible recognition, increased image variability during encoding simply reduces the extent to which flexible recognition is required to successfully recognize the person. Thus, work to date suggests that perhaps the only consistent way to improve recognition for changed stimuli is to increase the similarity between a studied stimulus and the tested stimulus to reduce the magnitude of the change.

While it is clear that increasing similarity between encoding and retrieval is the best identified method for overcoming the problem of poor recognition for changed stimuli, it is not clear how this could be broadly applied to real-world contexts such as eyewitness memory in which increasing actual study-test similarity is often not possible. We propose that it might be possible to *simulate* an increase in study-test similarity using mental imagery. Using the previously described Read et al. (1990) aging faces study as an example, participants’ memory representations could perhaps be rendered less rigid during encoding by asking participants to imagine what the faces would look like when older, to anticipate the test stimulus. It is well known that mental imagery can successfully simulate memories, and that imagery during encoding improves later memory (Bower, 1972; Bower & Winzenz, 1970; Hyman et al., 1996; Lampinen et al., 2003; Loftus & Pickrell, 1995; McCauley et al., 1996; G. Murphy et al., 2023; Paivio & Csapo, 1973). In fact, imagining a scene immediately after viewing it during encoding produces equivalent levels of recognition to actually seeing the scene again (Yi et al., 2008). Even more striking, mental imagery can produce entirely new memory traces that can be similar in richness to veridical memories (De Brigard et al., 2020; Devitt & Addis, 2016; Heaps & Nash, 2001; Lampinen et al., 2003; Loftus & Pickrell, 1995), and counterfactual mental imagery—imagining an alternative version of a memory—increases false memory for those alternative versions of the memory (Gerlach et al., 2014). Therefore, the robust ability of imagery to strengthen, modify, and create memory representations suggests that it could be leveraged to increase similarity between a memory trace and a stimulus that one anticipates encountering in the future.

One potential obstacle to the utility of imagery is that most of the literature on imagery–memory interactions to date has focused on information that can be verbalized, such as who was present in an episode or where it took place (Devitt & Addis, 2016). It is possible that imagery-induced memory representations might be too abstract or propositional to aid in many real-world contexts such as facial recognition, given that facial recognition relies largely on nuanced geometric information that cannot be verbalized (Ellamil et al., 2008; Jiang et al., 2006; O’Toole et al., 1998; Schyns et al., 2002). However, recent neural work suggests that the memories created via visual imagery are not simply abstract or conceptual in nature: Visual imagery produces neural activity in visual perception regions that is analogous to actual perception (Reddy et al., 2010; Yi et al., 2008). For example, Reddy et al. (2010) found that imagined object categories could be decoded from perceptual activity just as well as they could be decoded from imagery-related activity. Moreover, neural activity during visual memory and visual imagery is also highly overlapping (Addis et al., 2009; De Brigard et al., 2013; Slotnick et al., 2012). Notably, the vividness of mental imagery appears to be critical for how effectively it can simulate perception and memory: The similarity of neural activity between perception and imagery increases as imagery vividness increases (Dijkstra et al., 2017), and the effects of encoding imagery on later memory increase as imagery vividness increases (D’Angiulli et al., 2013; Gjorgieva et al., 2023a, 2023b). Therefore, although there is promising evidence that visual imagery can simulate perceptual representations and downstream memory, any effects of imagery on flexible recognition would likely depend on the vividness of that imagery.

In sum, prior work points to the possibility that visual imagery could be leveraged to improve flexible recognition, especially when that imagery is vivid. However, this has not been tested to our knowledge. In the present study, we tested whether counterfactual visual imagery during initial encoding could be used to simulate changes in study-test similarity—and therefore directionally influence participants’ ability to recognize people whose appearance has changed.

## Current research

We developed a novel visual imagery and stimulus change manipulation and deployed it within a general paradigm commonly used in studies of eyewitness memory. Participants encoded faces, and in a subsequent retrieval phase, provided alternative forced-choice judgments and recognition confidence for lineups of old and new faces. We used a within-subjects directional imagery manipulation during encoding and compared memory performance between retrieval faces that were congruent versus incongruent with the imagery. The only manipulation was whether imagery was in the same direction as the change that would later occur—such that the congruent and incongruent conditions were identical in terms of encoding—thereby controlling for other encoding effects such as depth of processing. It should be noted that this design was not aimed at determining whether imagery in general is superior to other encoding strategies, or whether it can improve memory for unchanged stimuli as well; rather, we sought to determine whether memory representations could be directionally modified by imagery in a way that mimics changes in study-test similarity along a specific dimension (in this case, facial expression).

To this end, participants viewed neutral faces at encoding and were asked to imagine each face in either a happy or angry expression (randomly intermixed across trials). We assessed participants’ trial-by-trial subjective imagery vividness to allow us to examine trials in which imagery was successful versus unsuccessful. In a later test phase, participants saw both studied and new faces—presented within three-face arrays—in either happy or angry expressions, such that half of the studied faces matched the imagined expression (congruent) and the other half did not (incongruent). Some arrays contained only new faces to provide a control condition in which (veridical) memory was absent entirely (i.e., target-absent lineups). Participants were asked to select the old face within each array—or to guess if they thought all the faces were new—and then to indicate their recognition confidence about whether the face was old or new.

In terms of potential outcomes, our primary prediction was that memory would be better for test faces whose expression was congruent rather than incongruent with the imagined expression. For example, imagining a neutral face in a happy expression during encoding should confer a memory advantage if that face is later presented in a happy expression relative to an angry expression (and vice versa for angry/angry being superior to angry/happy). Moreover, we expected this effect to be larger for more vividly imagined expressions (Gjorgieva et al., 2023a, 2023b). Together, this would indicate that visual imagery can be used to simulate an increase in study-test similarity and therefore manipulate flexible recognition.

## Experiment 1

### Experiment 1 method

#### Participants

A total of 180 undergraduate participants (mean age = 19.22 years, age range = 18–24 years; 93 male; 86% identified as White; 7% as Asian; 5% as Black or African American; 3% as American Indian, Alaska Native, or Pacific Islander; 1% as unknown, and 4% as more than one race) successfully passed pre-experimental attention checks and completed the experiment online for course credit. These pre-experimental attention checks, which we have used in prior work (Ramey & Zabelina, 2024; Ramey et al., 2022), required participants to select the correct answer option in response to the initial instructions. For example, within an instruction paragraph telling participants that they would need to keep the browser window maximized and disable browser extensions, there was an embedded attention check: “To demonstrate that you have read these instructions, select “no” (do not select “yes”) below”. The 52 participants who selected “yes”—indicating they did not read the instructions—were excluded a priori from being counted as having participated, so they were not included in sample size counts (i.e., they did not contribute to the *n* = 180 sample size above). Additionally, to further improve data quality, 31 participants’ data were eliminated from analysis for failing attention checks throughout the experiment (25 participants), having below chance forced-choice accuracy or old/new recognition accuracy (20 participants), or making a face selection on < 75% of trials (i.e., they did not make a decision within the 10 s time limit; 18 participants). Attention checks throughout the experiment included additional embedded instructions, as well as passing the test of understanding for the memory scale (see below). Note that many participants who were excluded based on one criterion were also excluded based on at least one other. We have used these exclusion criteria and attention checks (including the pre-experimental attention checks) in prior work (Ramey et al., 2024) and find that they yield results equivalent to in-person data using the same task (with minimal numbers of participants needing to be excluded based on these criteria in person; Ramey & Lampinen, 2025). Additionally, 8 participants’ data (most of whom were already also excluded based on the other exclusion criteria) were removed because they indicated in a post-experiment debriefing question that they had not made an effort to conduct the visual imagery as instructed or had given up during the task; participants were informed that their responses would not be linked to their identity, and that there would be no penalty based on their response.

After these exclusions, the final sample consisted of 149 participants. A power analysis based on the effect of imagery during encoding on subsequent memory strength obtained in a prior study (Diana et al., 2008), *d*_*z*_ =.56, indicated that this sample size of 149 participants provided well over 99% power to detect an effect of this size in a two-tailed t test. All participants provided informed consent prior to participating. All procedures were approved by the university Institutional Review Board.

#### Apparatus

The memory task was conducted online using JavaScript via jsPsych (de Leeuw, 2015). Participants were instructed to use a computer with a browser size of at least 800 × 600 px. The experiment would not begin if a participant’s browser size was less than 800 × 600 px, but the experiment allowed them to begin if the participant expanded their browser’s window size sufficiently (i.e., larger than 800 px in width and 600 px in height); this requirement precluded use of a smartphone.

#### Materials

Face stimuli were selected from the Chicago Face Database (CFD; Ma et al., 2015) and FACES database (Ebner et al., 2010), in which people were photographed in different expressions. Of the CFD faces, half were classified by the database as male, half as female, half as black, and half as white. The FACES database consisted of white faces evenly split between younger, middle-aged, and older, as well as male and female. We drew as many faces as possible from each database to maximize the total number of faces; the number of faces drawn from each database was determined based on the ability to construct demographically matched and database-matched three-face arrays (described in more detail below). A total of 300 face stimuli were used: 141 faces were drawn from the CFD, and 159 faces were drawn from the FACES database. The resulting stimulus set that we used in both experiments consisted of 48% male faces and 52% female faces, with 26% black faces and 74% white faces. (Note that only CFD included black faces, whereas the FACES database did not.) Of these 300 face stimuli, 80 were presented at study and test, and 220 were presented as new lures at test. Stimuli were counterbalanced across old/new status as well as imagined expressions and presented expressions. In the study phase, the faces were presented at 480 × 600 px. In the test phase, the three-face arrays were presented at 800 × 600 px.

Colored squares were used to cue participants as to which expression they should imagine during the study phase. Arbitrary colored cues were used instead of the words “happy” and “angry” to avoid the possibility of participants forming associations between the words and the faces. The color pairs were selected to be discriminable with all types of colorblindness (Jenny & Kelso, 2007). Specifically, the pairs were (1) brown (#994f00) and blue (#006cd1), and (2) pink (#d35fb7) and yellow (#fefe62). In the debriefing questionnaire, no participants reported difficulty in discriminating the color cues. Cues were counterbalanced, and participants saw one pair during the first half of the study phase and the other pair during the second half to mitigate potential effects of color.

During the test phase, participants were presented with 100 three-face arrays, 80 of which contained one old face from the study phase along with two new lure faces and 20 of which contained only new faces. To avoid potential effects of selection bias based on demographics or database origin, the faces in each array were matched on database of origin (and therefore lighting and background color), as well as all demographic information provided by the face databases: age, race, and gender. Therefore, all three faces in each test array were matched on demographic and contextual factors. The resulting effective lineup size, measured in terms of Tredoux’s *e* (Tredoux, 1998; Tredoux & Naylor, 2018), was 2.43 across all arrays (old and new arrays, i.e., target-present and absent), indicating that the lineups were not biased (Malpass, 1981; Mansour et al., 2017). However, note that unlike most eyewitness experiments, there were no predefined targets or innocent suspects guiding lineup construction; rather, all faces were treated equivalently until random assignment to old/new status, and a face could be old or new depending on the counterbalance.

#### Procedure

The experiment lasted approximately 45 minutes and consisted of a study phase followed by a test phase. Before each phase, participants were given instructions as well as practice trials to familiarize them with the procedure. Participants were also given a break midway through each phase, as well as between phases.

**Fig. 1:**
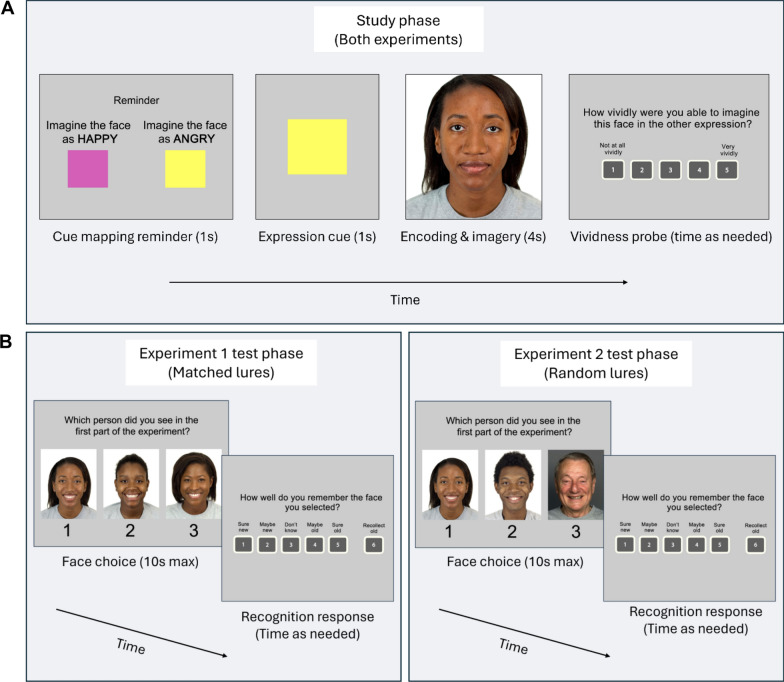
Stimuli and procedure. **A** The study phase procedure, in which participants encoded neutral faces and imagined them in a different expression. In each trial, participants were first shown a reminder of the mapping between the cue colors and expressions to be imagined; then, they were cued with one of the colors to indicate which expression they would be imagining for the upcoming face. A face in a neutral expression then appeared, and participants had 4 s to view the face and imagine it in the cued expression. After each face, participants reported the vividness of their imagery. Each face was shown once during the study phase and imagined in only one expression. The study phase was identical across Experiments 1 and 2. **B** The test phase procedure. In each trial, participants were shown an array of three faces, some of which contained an old (studied) face, and some of which contained only new faces. Participants were asked to select the old face—or, if they thought all the faces were new, to guess. Participants then provided a confidence-based recognition response to indicate if they thought the selected face (and therefore the array) was old or new. This trial is an example of an old array (i.e., contains a studied face) in the incongruent imagery condition, because the imagined expression (angry) did not match the tested expression (happy). The Experiment 2 test phase procedure was identical to Experiment 1, but the test arrays contained randomly selected lures (i.e., dissimilar lures), rather than lures matched on demographic information and database of origin (i.e., similar lures)

*Study Phase.* The study phase included 80 trials in which participants were cued, using colored squares, to imagine neutral faces in different expressions (Fig. 1a). Each face was shown only once and imagined in a single expression. The expression to be imagined was manipulated within subjects, with trials randomly intermixed. Participants were told that they would be shown faces, asked to imagine the faces in angry or happy expressions, and tested on their memory for the face identities later. They were not told that the memory test would be relevant to the imagined expressions, or that the stimuli would change at test. Participants were given two visual examples of angry and happy versions of sample neutral faces, to ensure that participants understood what was meant by the instructions to imagine faces in angry and happy expressions. Prior to beginning the task, participants were instructed and trained on the mapping between the colored square cues (i.e., *expression cues*) and their corresponding expressions.

Each trial first consisted of a 1 s screen reminding participants of the mapping between the expression cues and their corresponding expressions, followed by a 1 s expression cue. After the expression cue, a neutral face was presented for 4 s, and participants were tasked with spending this 4-s encoding period imagining the face in the cued expression. After the 4 s, a vividness probe appeared, in which participants were asked to rate how vividly they were able to imagine the face in the other expression on a 1–5 scale.

*Test Phase*. After the study phase, participants were given a 2-min break, and then the test phase began (Fig. 1b). The test phase consisted of 100 trials in which participants were asked to select the old face in a three-alternative forced-choice test, as well as to report their recognition confidence for each array. Specifically, participants were given 10 s to use their keyboard to select which face in each three-face array they remembered from the study phase, or to guess if they did not remember any. Then, participants were asked to report their memory confidence for the selected face using their keyboard; if they thought that none of the faces were old, they were asked to respond that the face was new. Of the 100 three-face arrays, 80 contained a studied face and two new (lure) faces and 20 consisted only of new faces. Importantly, none of the faces were presented in a neutral expression at test. Rather, half of the arrays contained faces in an angry expression, and the other half contained faces in a happy expression; within a given array, all faces had the same expression. Thus, half of the old faces matched the expression in which they were imagined at study (congruent), whereas the other half did not match the expression in which they were imagined at study (incongruent). At test, within each congruency condition, half of the old faces were presented as angry and half were presented as happy.

After selecting a face, participants gave their recognition confidence response by rating memory confidence on a 6-point scale that has been extensively validated in prior work (Ramey & Zabelina, 2024; Ramey et al., 2025; Yonelinas et al., 2022). These responses were used for computing memory performance via AUC. Response options included “Recollect old (6),” “I’m sure it’s old (5),” “Maybe it’s old (4),” “I don’t know (3),” “Maybe it’s new (2),” and “I’m sure it’s new (1).” Participants were instructed and tested on how to use this scale prior to beginning the test phase. Specifically, they were given detailed instructions on the definitions of old and new, and what each confidence response referred to. Participants were then given a test of understanding for the scale, which they had to pass to proceed. This test included a series of examples such as “if you think you might have seen the face before, but you aren’t sure, which response should you select?” (the answer being 4, “maybe old”).

#### Data reduction and analysis

*Examining Trials in Which Imagery Succeeded*. Given that imagery is an effortful process, we were particularly interested in examining the effects of the imagery congruency manipulation on trials for which imagery was successful. Based on prior work (Gjorgieva et al., 2023a, 2023b), we expected that any effects of the imagery manipulation would primarily, if not selectively, affect faces for which participants reported high imagery vividness. To index successful imagery, we examined trials in which vividness ratings were greater than three (i.e., on the upper half of the scale, which ranged from one to five); this cutoff was selected to include approximately half the data, in that 52.7% of trials were retained with this cutoff. However, note that analyses are first reported with all trials included, and we also report how effects varied across the continuum of vividness ratings.

*Statistical Models*. When memory performance was compared to chance, a one-sample t test was used. All other statistical analyses were conducted using multilevel modeling. For analyses of data averaged by subject (i.e., AUC and alternative forced-choice accuracy), random intercepts of subject were used. For analyses of trial-level data (i.e., recognition responses and vividness responses), crossed random intercepts of subject and stimulus were used. We also tested random slopes for each analysis in which slopes could be identifiable—allowing the predictor(s) of interest to vary by subject (and stimulus when applicable)—but found that most models were overparameterized (i.e., singular fits) when random slopes were included. Nonetheless, the pattern of results was unchanged when random slopes were included: Significant effects remained significant, and nonsignificant effects remained nonsignificant (with one exception noted in the results for Experiment 2). For continuous and binary outcomes, the models were estimated using the *lmerTest* package in R (Kuznetsova et al., 2017). For ordinal outcomes, the models were estimated using the *ordinal* library in R (Christensen, 2023). Effect sizes are reported as the standardized model estimate for the variable of interest (i.e., when all numeric parameters are scaled); that is, β for continuous fixed effects (*B* when the outcome was binary) and standardized mean difference (*SMD*) for categorical fixed effects.

To quantify the evidence in favor of the null for key nonsignificant results obtained using frequentist statistics, we computed Bayes factors (using the *BayesFactor* package in R with 5000 iterations; Morey et al., 2015) and assessed BF_10_—that is, the ratio of the Bayes factor in favor of the alternative hypothesis to the Bayes factor in favor of the null hypothesis. By convention, a BF_10_ < 0.33 indicates moderate evidence for the null hypothesis, and a BF_10_ < 0.10 indicates strong evidence for the null (Lee & Wagenmakers, 2013).

*Data and Code Availability*. The data and code for both experiments are available on OSF: https://osf.io/jnkfw/?view_only=3327b64d37f14a6eb46d87080758a63f

### Experiment 1 results

#### Old/new recognition performance

We first examined old/new recognition performance for the arrays. That is, we calculated area under the curve (AUC) in terms of correctly discriminating old arrays (i.e., arrays that contained an old face) from new arrays (i.e., arrays that contained only new faces) based on the recognition confidence responses. For old arrays, recognition confidence was only analyzed for trials in which participants correctly selected the old face (but see below for analysis with all trials). AUC was well above chance, *t*(148) = 22.8, *p* <.001, indicating that participants successfully discriminated old from new faces.

Recognition performance was modulated by the imagery manipulation such that AUC was higher when the old face was congruent rather than incongruent with the imagined expression, *SMD* =.20, *SE* =.07, *p* <.001. Converging with this, at the trial level, recognition confidence was higher for congruent than incongruent old arrays, *SMD* =.13, *SE* =.03*, p* <.001. Surprisingly, however, there was no interaction by face selection accuracy, *SMD* =.04, *SE* =.07, *p* =.60, with strong Bayesian evidence for the null, BF_10_ =.03. That is, the influence of imagery congruency on recognition confidence in old arrays was similar irrespective of whether participants correctly selected the old face or incorrectly selected a new face. When examined separately based on face selection, congruent imagery produced significantly higher recognition confidence both for trials in which the old face was correctly selected, *SMD* =.16, *SE* =.05, *p* <.001, and for trials in which a lure face was selected, *SMD* =.13, *SE* =.05, *p* =.008, with a quite similar magnitude of effects (i.e., *SMD* =.16 vs .13). These results therefore indicate that congruent visual imagery increased confidence that an old face was present in old arrays—even when participants could not tell which face in the array was old.

**Fig. 2:**
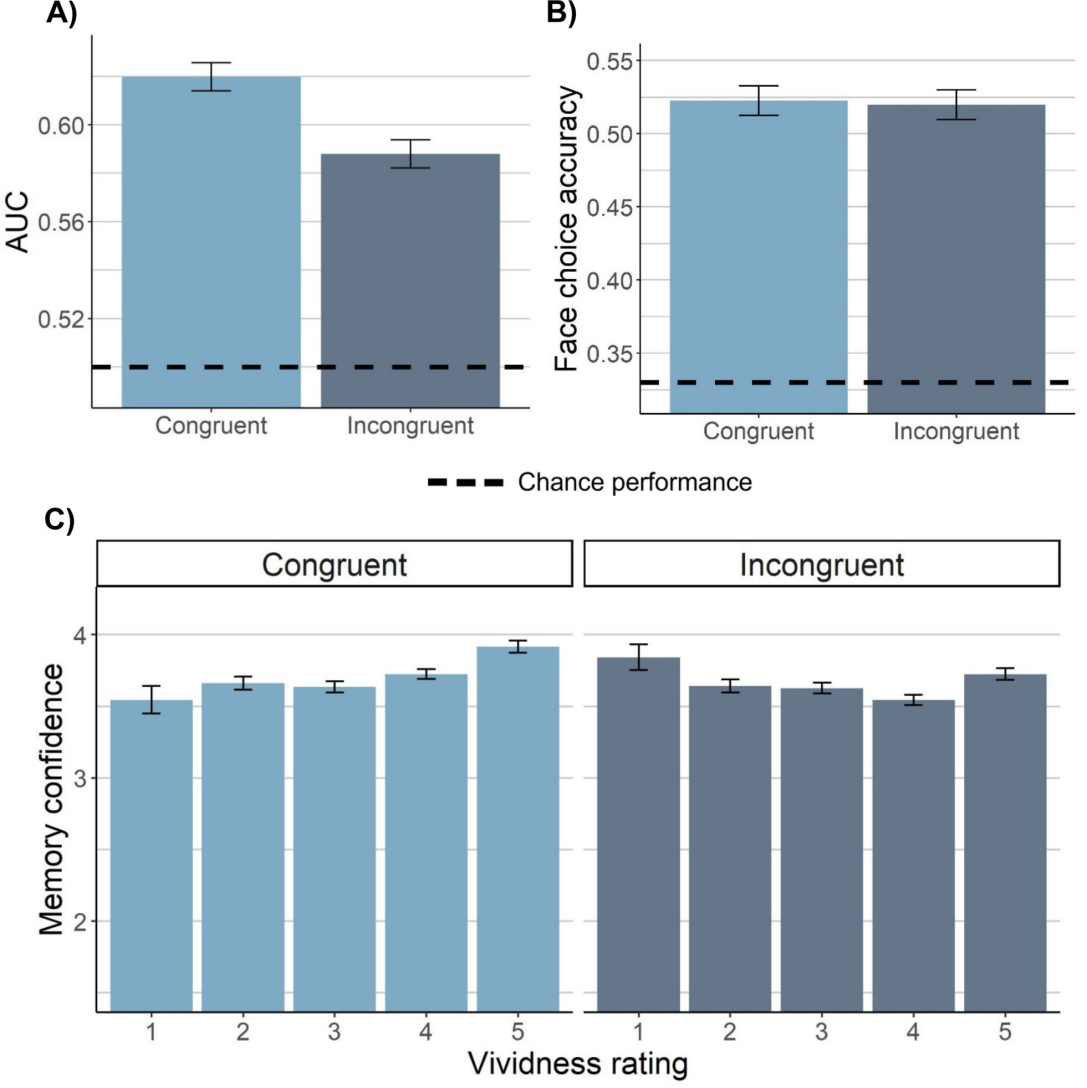
Effects of imagery manipulation on memory performance. Congruency refers to whether a face’s imagined expression during study matched the expression in which it was presented at test. **A** Old/new recognition performance in terms of AUC (including all old arrays) by congruency condition for vividly imagined faces. **B** Face-choice accuracy by condition for vividly imagined faces. (Note that when all faces are included, regardless of vividness, the numerical trend is in the opposite direction as AUC.) **C** The relationship between vividness and average recognition response by condition. For all plots, the estimated marginal means derived from a linear mixed effects model with random intercept of subject (and stimulus for plot C, given that it was not subject-averaged) are plotted, and the error bars represent the within-subject standard error of these estimated means from the model

To follow up on this unexpected effect, we reanalyzed AUC by again comparing old arrays to new arrays—but in this case, only including old arrays in which participants incorrectly selected the lure face. AUC was higher for congruent than incongruent expressions, *SMD* =.19, *SE* =.08, *p* =.02, with a nearly identical effect size to AUC calculated with correct selections only (i.e., *SMD* =.20; see above). There was no interaction by selection type with respect to AUC, *SMD* =.02, *SE* =.10, *p* =.83. Examining AUC with all old arrays included (no longer considering face selection) mirrored these findings, *SMD* =.22, *SE* =.06, *p* <.001 (Fig. 2a). Thus, these results corroborate the recognition confidence findings above and extend them to indicate that congruent visual imagery improved memory discriminability for old versus new arrays irrespective of the ability to determine which face was old.

Overall, our main hypothesis was supported in that AUC and recognition confidence were higher for congruent compared to incongruent faces. Surprisingly, however, this effect was not contingent on selection of the correct face. Rather, it appears that the effect of congruent visual imagery on recognition involved a diffuse sense of recognition that was present irrespective of the ability to correctly identify which face was old.

#### Face-choice accuracy

Next, we computed face-choice accuracy within old arrays. Participants were well above chance (*M* =.51; chance =.33) in selecting the old face in old arrays, *t*(148) = 23.2, *p* <.001, and face-choice accuracy was moderately correlated with recognition AUC across participants, *r* =.47, *p* <.001. Interestingly, however, face-choice accuracy was not affected by imagery congruency, *SMD* = -.01, *SE* =.09, *p* =.88, with moderate Bayesian evidence for the null effect, BF_10_ = 0.12 (Fig. 2b). Not only was the effect nonsignificant, but in terms of numeric trends, the effect was in the opposite direction of the AUC effects: Forced-choice accuracy was numerically poorer for congruent than incongruent faces. The lack of effect of imagery on face-choice accuracy was also found at the trial level in a logistic mixed effects regression, *B* = −.01, *SE* =.04, *p* =.86. Therefore, although imagery congruency influenced memory in terms of recognizing old arrays, it did not influence memory in terms of the ability to select the old face within the old arrays. Thus, the results of face-choice accuracy were not in line with what we expected: Congruent visual imagery did not improve participants’ ability to select the studied face in a lineup.

#### Vividness

We next examined the effects of participants’ reported vividness of their visual imagery for each face during encoding. As predicted, the effect of imagery congruency on recognition confidence was stronger for more vividly imagined faces, such that the effect of congruency on recognition confidence for old arrays linearly increased with vividness, *β* =.14, *SE* =.03, *p* <.001 (Fig. 2c). This indicates that the influence of congruent imagery on old/new recognition is stronger with more vivid imagery.

We next reanalyzed the AUC and face-choice effects including only trials with vividness scores greater than three (i.e., the most vivid 53% of trials), to ensure that effects held when only the most successful imagery trials were examined. The effect of imagery congruency on overall AUC held—and was in fact approximately 50% stronger—when only the more vividly imagined faces were examined, *SMD* =.31, *SE* =.08, *p* <.001. The lack of effect of imagery on face-choice accuracy also held when only the vividly imagined faces were examined, *SMD* = −.02, *SE* =.10, *p* =.85, including the moderate evidence for the null effect, BF_10_ = 0.13. The null effect of imagery congruency on face-choice accuracy did not vary with vividness, *B* =.04, *SE* =.04, *p* =.26.

Additional descriptive statistics, as well as analyses by expression type, are available in the Supplementary Materials.

### Experiment 1 discussion

In Experiment 1, we tested a novel manipulation aimed at influencing the ability to recognize a person whose appearance has changed. During encoding, participants viewed neutral faces and used visual imagery to simulate the faces in different expressions. Overall, we found that visual imagery directionally influenced memory based on whether the imagery was congruent or incongruent with the expression in which a given face was presented during later retrieval. Our hypotheses were confirmed in that recognition performance was better for congruent than incongruent imagery, and this effect increased with increasing imagery vividness. These findings suggest that visual imagery can be used to simulate changes in study-test similarity. Unexpectedly, however, the effects were limited to recognition of the arrays as a whole in that forced-choice accuracy was not affected. That is, congruent imagery increased the likelihood that participants successfully recognized an old array as containing an old face (compared to all-new arrays), without improving participants’ ability to determine which face in the old arrays was old. Together, these findings suggest that directional imagery during encoding alters memory representations in a way that influences a global, diffuse sense of recognition without influencing the ability to attribute that sense of recognition to a specific aspect of a test stimulus.

The fact that old/new recognition performance was dissociated from forced-choice accuracy is surprising, but it is not unprecedented. This dissociation has previously been found such that forced-choice accuracy and old/new recognition are associated with distinct neural signatures and can be influenced in opposite directions by behavioral manipulations (Voss & Paller, 2009, 2010; Voss et al., 2008, 2012). The prevailing interpretation of these effects (see commentaries appended to Voss et al., 2012) is that forced-choice accuracy is primarily based on unconscious memory processes such as fluency, whereas conscious recognition is often based on other processes such as global similarity computations. Our findings add to this by suggesting that the type(s) of memory that are unrelated to forced-choice accuracy can be selectively manipulated, in this case by directional visual imagery during encoding. Thus, the present results provide a complementary dissociation to the dissociations observed by Voss et al. (2008), Voss & Paller (2009).

Importantly, the conditions of the present study are largely in line with what Voss and colleagues propose as being optimal for revealing dissociations between old/new recognition and forced-choice accuracy (Voss & Paller, 2009, 2010; Voss et al., 2008, 2012). First, conceptual information was minimal given that the stimuli were faces, such that encoding was likely based primarily on perceptual information rather than elaborative encoding strategies. Second, guessing on face selection was explicitly encouraged. Third, and most robustly established as an important condition for revealing dissociations, there was a high degree of similarity between the lures and the old faces in the test arrays (see Fig. 1b). That is, to avoid selection biases (i.e., to construct fair lineups; Smith et al., 2017; Wixted & Mickes, 2014), each test array consisted of three faces that were matched in terms of age, race, and gender, as well as database of origin (and therefore lighting and background color). This design was used to render the paradigm and results directly applicable to understanding eyewitness memory effects, given that (fair) lineups consist of individuals matched on these traits.

Despite the advantages of using matched lures in our paradigm, prior work indicates that reversals of typical memory effects can occur when highly similar lures are used. For example, confidence and accuracy in alternative forced-choice are typically strongly correlated; however, when highly similar lures are used under some conditions, accuracy and confidence can become dissociated (Heathcote et al., 2009, 2010; Tulving, 1981). Moreover, when lures are made more similar to the target, forced-choice accuracy suffers but confidence is often unaffected (Hanczakowski et al., 2021). Therefore, the lack of effect of imagery on forced-choice accuracy in the present experiment may have been a result of high lure similarity at retrieval.

We ran a second experiment to (1) attempt to replicate the effects observed in Experiment 1 and (2) test the possibility that lure similarity in the retrieval arrays drove the dissociation between old/new recognition and forced-choice accuracy. In Experiment 2, lure faces were randomly selected for each test array, without any demographic or database matching. If the dissociations between forced-choice accuracy and recognition performance were driven by high lure similarity within the arrays, then the dissociations should not be found when dissimilar lures are used. That is, imagery should influence both old/new recognition and forced-choice accuracy. In contrast, if the dissociations were not driven by high lure similarity, then the pattern of effects from Experiment 1 should be replicated even when dissimilar lures are used.

## Experiment 2

### Experiment 2 method

The apparatus, procedure, and data reduction and analysis were identical to Experiment 1. The only difference in Experiment 2 was that faces in each test array were not matched on any characteristics; rather, the lures in each test array were randomly selected (Fig. 1b). Specifically, all faces—regardless of database or demographics—were combined into a single pool that was randomly selected from when making the test arrays. This rendered the faces within each test array less similar. Note that, as in Experiment 1, old and new status of faces was counterbalanced such that a given face would serve as an old face for some participants and a new face for others. The resulting effective lineup size, measured in terms of Tredoux’s *e* (Tredoux, 1998; Tredoux & Naylor, 2018), was 2.43 across all arrays (old and new arrays, i.e., target-present and absent), indicating that the lineups were not biased (Malpass, 1981; Mansour et al., 2017).

Moreover, as expected, the influence of the imagery manipulation in Experiment 1 depended on the vividness of that imagery—that is, imagery primarily exerted effects on memory when participants reported that imagery was successful. In Experiment 2, we also ran follow-up analyses focusing on more vividly imagined trials using the same criteria as Experiment 1: vividness responses greater than three, which included 56% of trials (but we first present analyses with all trials included).

#### Participants

A total of 208 undergraduate participants (mean age = 18.88 years, age range 18–27 years; 147 female; 90% identified as White; 4% as Asian; 3% as Black or African American; 3% as American Indian, Alaska Native, or Pacific Islander; 1% as unknown, and 6% as more than one race) successfully passed pre-experimental attention checks and completed the experiment for course credit. Sixty-one participants failed pre-experimental attention checks; as in Experiment 1, these participants were not counted toward the *n* = 208 sample size. Participant data (*n* = 40) were eliminated from analysis based on the same criteria as Experiment 1: failing attention checks throughout the experiment (36 participants), having below chance forced-choice accuracy or old/new recognition accuracy (27 participants), or making a face selection on < 75% of trials (i.e., they did not make a decision within the 10 s time limit; 12 participants). Moreover, 10 participants’ data (many of whom were already also excluded based on the other exclusion criteria) were removed because they indicated in a post-experiment debriefing question that they had not made an effort to conduct the visual imagery as instructed or had given up during the task. After these exclusions, the final sample consisted of 168 participants. A power analysis based on the effect of imagery congruency on AUC for vivid trials in Experiment 1, *d*_*z*_ =.31, indicated that a sample size of 168 participants provided 98% power to detect the effect. All participants provided informed consent prior to participating. All procedures were approved by the university Institutional Review Board.

### Experiment 2 results

#### Old/new recognition performance

First, we examined effects when trials of all vividness levels were included. Participants were above chance in discriminating old from new arrays in terms of AUC, *t*(167) = 15.86, *p* <.001. Overall AUC (including old and new arrays) was .60, equivalent to Experiment 1 (also .60). The effect of congruency on AUC (in which only old arrays with correct face selections were included) was in the same direction as Experiment 1 but was not significant, *SMD* =.12, *SE* =.08, *p* =.11. However, with all old arrays included regardless of face selection, AUC was significantly better for congruent than incongruent imagery, *SMD* =.13, *SE* =.06, *p* =.04, replicating the Experiment 1 effect.

At the trial level, recognition confidence in old arrays was higher for congruent than incongruent faces, *SMD* =.09, *SE* =.03, *p* =.002 (though note this was no longer significant with random slopes included, *p* =.14). There was again no interaction with face selection, such that congruency effects on old/new recognition confidence did not differ for correct versus incorrect selections, *SMD* =.05, *SE* =.06, *p* =.48.

**Fig. 3:**
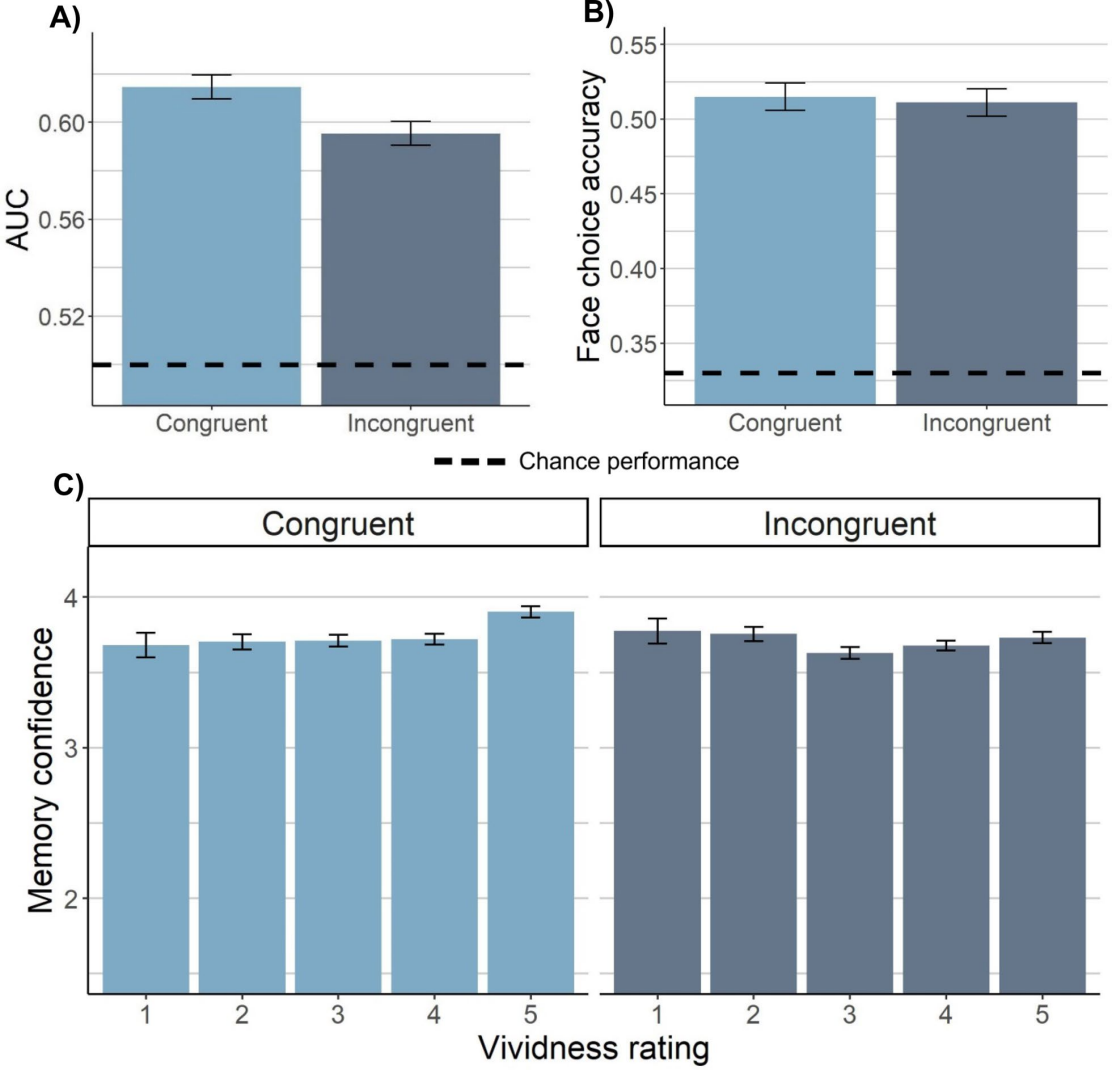
Experiment 2 results. **A** Old/new recognition performance in terms of AUC (including all old arrays) by congruency condition for vividly imagined faces. **B** Face-choice accuracy by condition for vividly imagined faces. **C** The relationship between vividness and average recognition response by condition. For all plots, the estimated marginal means derived from a linear mixed effects model with random intercept of subject (and stimulus for plot C, given that it was not subject-averaged) are plotted, and the error bars represent the within-subject standard error of these estimated means from the model

The effect of vividness on the congruency effect was replicated: The benefits of congruency for old/new recognition confidence were higher when vividness was higher, *β* =.09, *SE* =.03, *p* =.003 (Fig. 3c).

Overall, these results indicate that the imagery manipulation increased AUC for congruent imagery, but that the effects were not as robust in Experiment 2 as in Experiment 1 (but see analyses by vividness below).

#### Face-choice accuracy

Participants exhibited significantly above-chance forced-choice accuracy, *t*(167) = 25.87, *p* <.001. Forced-choice accuracy was .48, similar to Experiment 1 (.49). Even with dissimilar arrays at test, the lack of effect of imagery congruency on face-choice accuracy was also replicated, *SMD* =.08*, SE* =.08*, p* =.32, again with moderate Bayesian evidence for the null, BF_10_ = 0.19.

Therefore, the effects obtained were similar to Experiment 1 in that imagery influenced old/new recognition performance without influencing forced-choice accuracy.

#### Vividness

Next, we examined trials in which imagery was successful. As in Experiment 1, congruent imagery significantly increased AUC when only correct face selections were included for old arrays, *SMD* =.19, *SE* =.07, *p* =.03, as well as when all old arrays were included, *SMD* =.19, *SE* =.07, *p* = *.*007 (Fig. 3a). Congruent visual imagery increased recognition confidence for old arrays overall, *SMD* =.15, *SE* =.04, *p* <.001, and there was again no interaction by correct versus incorrect face selection, *SMD* =.03, *SE* =.09, *p* =.72, with strong Bayesian evidence for the null, BF_10_ =.04. The lack of congruency effect on forced-choice accuracy remained, *SMD* =.03, *SE* =.09, *p* =.77, again with moderate Bayesian evidence for the null, BF_10_ = 0.12 (Fig. 3b). Therefore, the results from Experiment 1 were fully replicated in Experiment 2 trials in which imagery was successful: Congruent visual imagery at encoding improved AUC and recognition confidence, and this was not dependent on selecting the correct face.

Overall, these findings suggest that the effects of congruency on recognition performance were less robust to low vividness in Experiment 2 than Experiment 1. That is, the effects of imagery congruency on recognition appeared to depend more on imagery success in Experiment 2. The results of Experiment 2 thus suggest that the effects of congruency on recognition remain even when dissimilar lures are used in the test arrays, but these effects are weaker or more dependent on imagery success.

Additional descriptive statistics, as well as analyses by expression type, are available in the Supplementary Materials.

### Experiment 2 discussion

The results of Experiment 2 replicated the key effects from Experiment 1. We again found that visual imagery during encoding directionally influenced recognition for changed faces. Specifically, relative to incongruent imagery, congruent imagery selectively improved old/new recognition for the arrays. There was substantial Bayesian evidence for a lack of improvement to forced-choice accuracy. One difference between experiments, however, was that the effects of Experiment 2 appeared to be weaker or more contingent on vividness. That is, Experiment 2 effects were less robust than the Experiment 1 effects when encoding trials in which visual imagery was unsuccessful were included. Overall, the results indicate that the dissociation between old/new recognition and forced-choice accuracy was not solely driven by the use of highly similar lures in the test arrays. Rather, these effects remained even when dissimilar lures were used.

Interestingly, despite the use of less similar test arrays, AUC and forced-choice accuracy were very similar between experiments. One possibility is that cohort effects, such as the semester in which data collection occurred, produced lower performance in Experiment 2 that counteracted any benefits from reduced lure similarity. Another possibility is that the similarity of the lures at test was not a significant driver of performance in this task. Further supporting this, lineup fairness (in terms of Tredoux’s *e*) was also very similar between studies. It is possible that other types of similarity that could not be systematically controlled for across participants, such as subjective similarity between specific pairs of faces, influenced performance. It is also likely that the similarity of encoded faces, irrespective of which were presented as lures during test, was a driver of performance. Regardless, these results suggest that the pattern of effects we obtained was not driven by similarity effects of lures in a given array at retrieval.

## General Discussion

In the present study, we tested the effects of visual imagery on flexible recognition: the ability to recognize an entity across perceptual changes that occur between encoding and retrieval. We designed a novel manipulation using visual imagery to directionally alter encoded memory representations, with the goal of influencing the ability to later recognize faces that had changes to their appearance. Across two experiments, participants encoded neutral faces while imagining them in either happy or angry expressions (manipulated within-subjects), and in a later test phase, participants were presented with lineups of new and studied faces in either happy or angry expressions (also manipulated within-subjects). We found that when the tested expression was congruent with the imagined expression—for example, when a face was imagined with a happy expression and then later presented with a happy expression at retrieval—old/new recognition performance was better and confidence was higher than when the tested expression was incongruent with the imagined expression. This memory difference between congruent and incongruent imagery increased with increasing reported imagery vividness. Interestingly, however, alternative forced-choice accuracy was not influenced by imagery congruency, in that participants’ ability to select the old face in an array of three faces did not differ with congruency. This surprising set of findings was replicated in a second experiment, which further indicated that the effects were not due to high similarity between the target and the lures in the test arrays. These findings indicate that visual imagery during encoding can manipulate later flexible recognition in terms of the ability to recognize the presence of a previously seen person, but not the ability to determine which person is familiar. Thus, these results indicate that visual imagery during encoding can, unexpectedly, robustly dissociate old/new recognition from forced-choice accuracy.

The present study was motivated by an issue that plagues eyewitness memory and missing persons identification: the fact that we are, on average, quite poor at recognizing people across even minor changes in their appearance (Young & Burton, 2017, 2018). Our results indicate that using visual imagery to simulate an increase in study-test similarity could perhaps have some utility in specific contexts. With respect to eyewitness memory, our results suggest that visual imagery could be useful for showups, in which a witness is presented with a single suspect and asked to determine whether or not they were the perpetrator (Steblay et al., 2003). It is established that lineups are the optimal procedure for minimizing false identifications, but showups are still very commonly used in practice (Wells et al., 2020). Our findings indicate that visual imagery could potentially be leveraged to improve showup accuracy by improving overall old/new memory discriminability. For example, if a witness observes a perpetrator committing a crime while wearing dark colors and under minimal lighting, our findings suggest that the witness’ later showup identification accuracy could potentially be improved by imagining the perpetrator under police station lights in more standard clothes prior to the showup. Of course, future studies are needed to confirm this, as the present studies did not include a single-face condition that would be directly applicable to showups (but see Ramey & Shields, 2025).

Our findings also carry implications for techniques that are used to allow witnesses to reconstruct perpetrators from memory (e.g., software versions of forensic sketch artists). Specifically, the present findings—along with other studies demonstrating the impact of facial expression changes on recognition memory (D’Argembeau & Van Der Linden, 2011; D’Argembeau et al., 2003; Ellamil et al., 2008; Vakil et al., 2019)—suggest that software that is used to allow eyewitnesses to reconstruct perpetrator faces (e.g., composite construction systems) may benefit from inclusion of facial expression. At least one composite construction system that tends to outperform other systems, EvoFIT, already includes holistic dimensions such as subtle structural changes in how threatening a face is (Frowd et al., 2006, 2015); a more dramatic dimension of expression may further improve witnesses’ ability to accurately reconstruct faces from memory. With respect to missing persons identification, an external analogue of our manipulation is already in use: age progression to create updated pictures of what missing persons might currently look like (Lampinen et al., 2015, 2016). Our findings suggest that visual imagery may be able to act as a weaker, but perhaps more broadly applicable, version of this. Therefore, further investigation of the real-world utility of the present flexible recognition manipulation is warranted and may yield encoding strategies to improve eyewitness memory and prospective person memory.

With respect to theory, the present findings bear on models of eyewitness memory. A prevailing class of models that has been used to characterize eyewitness memory processes—global matching models—proposes that lineup decisions are based on a continuous memory signal that results from comparing the presented faces with an internal representation of the perpetrator (Clark & Gronlund, 1996; Mickes et al., 2025). These models generally propose that lineup decisions depend on both the absolute match of the best candidate (i.e., most familiar-seeming face) to memory as well as the relative match of the best candidate over the other lineup candidates (Clark, 2003; Clark et al., 2011; Moreland & Clark, 2020). Under this framework, our findings could perhaps be interpreted as the visual imagery manipulation increasing absolute match (i.e., the ability to determine that an old face is present or absent in the array) but not increasing relative match of the old relative to lure faces (i.e., the ability to determine which face is old). Our results also carry implications for formal definitions of absolute match. Current models, such as the WITNESS model, tend to model absolute match as the match of the best (most familiar) lineup member to memory (Clark, 2003; Moreland & Clark, 2020): If the level of match is above a certain criterion, an identification is made (or, in the context of our experiment, participants make an “old” response). Our findings could be taken to imply that absolute match may not in fact involve a (consciously available) sense of which lineup member provides the best match; rather, it could be a global sense of match that cannot be attributed to a specific lineup member. This could perhaps explain the conflicting results that have been obtained in both empirical and modeling investigations attempting to understand absolute versus relative match (Clark et al., 2011; Moreland & Clark, 2020). We are currently examining the memory processes underlying the present effects, to assess whether they are driven by changes in matching processes (more broadly defined) versus more active retrieval processes (Ramey & Shields, 2025).

These results also have important implications for theories of recognition memory more broadly. Currently, the prevailing view is that old/new recognition and forced-choice accuracy rely on largely the same processes. However, some studies have been able to manipulate them separately (Voss & Paller, 2009; Voss et al., 2008), suggesting that the processes underlying each type of memory test may be partially dissociable. In both of our experiments, we observed a novel complementary dissociation in which the imagery manipulation influenced old/new recognition without influencing forced-choice accuracy. Importantly, the lack of effect on forced-choice accuracy was not simply a nonsignificant effect: The numerical trend was in the opposite direction of old/new recognition, and there was Bayesian evidence for the null. When combined with prior findings, these results provide evidence for a double dissociation between forced-choice accuracy and old/new recognition. This indicates that current theories of recognition memory need to be updated to account for these dissociations.

The present study also informs our understanding of imagery-memory interactions. As outlined previously, studies of the influence of imagery on memory have primarily focused on verbalizable information such as the components of a memory (e.g., who was present, where it took place, and what events occurred; Devitt & Addis, 2016). The extent to which imagery can be used to influence memories for complex perceptual information has been less clear. The present results suggest that imagery can be used to modify memory encoding and manipulate later recognition for previously unfamiliar faces, which depends on nuanced perceptual differences in face geometry that generally cannot be verbalized. Therefore, our findings provide a behavioral validation of what has been suggested by neural studies: that visual perception and visual imagery are highly overlapping (Reddy et al., 2010; Slotnick et al., 2012). However, the fact that visual imagery produced dissociable effects on old/new recognition and forced-choice accuracy indicates that there are nuanced differences in how visual imagery versus perception influence memory. Future studies probing these potential differences, in addition to those finding distinct effects on subjective experience (Devitt & Addis, 2016; Lampinen et al., 2003; Özbek et al., 2017), may be particularly fruitful for our understanding of imagery and memory.

One consideration for interpreting and generalizing these results is that the ability to successfully simulate an increase in study-test similarity depends on the ability to predict what the test stimulus may look like. This almost certainly depends on pre-experimental episodic memory and semantic knowledge (Addis et al., 2009; Devitt & Addis, 2016; Erickson et al., 2023; Irish, 2016, 2020; Schacter & Addis, 2007); for example, a lifetime of experience with facial expressions allows us to imagine what a face may look like in a different expression. Thus, successful imagery likely could not occur without relevant preexisting episodic memory or semantic knowledge. A second consideration with respect to generalizability is the extent to which these findings are limited to faces. Prior work suggests that changes between encoding and retrieval, such as changes in viewpoint, lead to indistinguishable behavioral effects for faces and objects (Ashworth III et al., 2008; Brooks et al., 2002). It is therefore possible that visual imagery could be used to bolster the encoding of flexible, abstracted representations across a range of stimuli, but future studies are needed to confirm this. A third consideration is how effects may differ depending on the number of trials. We used many trials to achieve sufficient power for this initial investigation, but it is possible that using fewer trials—such as in real-world contexts—could yield different effects. For example, effects could be stronger when there is less interference and less cognitive fatigue from prolonged imagery.

There are limitations to the present study that should be noted. First, assessing both forced-choice performance and recognition confidence within the same task is not a standard method outside of the eyewitness memory and metamemory literatures. However, this procedure has been leveraged in other paradigms aimed at examining basic recognition processes (Voss & Paller, 2009, 2010; Voss et al., 2008, 2012). The use of both forced-choice decisions and recognition confidence may render our results less generalizable to standard old/new recognition paradigms (Lampinen, 2016), because the recognition responses are partially contingent on the face selection in terms of the instructions given to participants. Interestingly, however, the effects we observed are in the opposite direction of what one would expect from spurious effects of contingent selection: Participants still had above-chance recognition for the old arrays overall even when selecting the wrong face, indicating that participants’ sense of recognition was not simply contingent upon selecting the correct face. A second limitation is that although our manipulation demonstrated that congruent imagery was better than incongruent imagery for recognition, our findings cannot speak to how these conditions compare to other encoding strategies. Moreover, because all faces were changed, it is not yet clear how the visual imagery manipulation may influence memory for unchanged faces, or faces changed in other ways not relevant to the imagery. Instead, for the present investigation, we focused on whether memory could be made more flexible in a specific direction, rather than more flexible overall. A third limitation is that our findings cannot definitively establish whether lure-target similarity influenced the presently identified effects. Specifically, although the effects were of smaller magnitude and more dependent on high imagery vividness in Experiment 2—in which dissimilar lures were used in the test arrays—we did not manipulate similarity within a single study. Our focus was instead on determining whether the effects replicated even when dissimilar lures were used. A related issue is that it is possible that the similarity between all of the encoded stimuli, rather than the specific lures presented alongside a given target, may be partially responsible for the present effects. That is, using a smaller or more heterogeneous stimulus set may alter the pattern of effects. A potential moderating role of similarity among encoded stimuli is an interesting possibility that should be investigated in future studies designed for that purpose.

## Conclusion

In sum, our findings indicate that counterfactual visual imagery during encoding can be used to simulate the effects of higher study-test similarity on old/new recognition, but not on forced-choice accuracy. That is, directed visual imagery appears to modify memory representations in a way that influences a diffuse sense of recognition, without the ability to attribute that recognition to a specific member of a lineup. Therefore, directed visual imagery could perhaps be used as an encoding strategy in some real-world eyewitness and missing persons contexts and may prove particularly useful to future studies aimed at understanding the shared versus distinct processes underpinning old/new recognition and forced-choice accuracy.

## Supplementary Information

Additional file 1.

## Data Availability

The data and code for the experiments are available on OSF: https://osf.io/jnkfw/?view_only=3327b64d37f14a6eb46d87080758a63f
