# Supplementary material for: Using visual imagery to manipulate recognition memory for faces whose appearance has changed
Source: Cogn Res Princ Implic. 2025 Sep 30;10:65. doi: 10.1186/s41235-025-00671-0 (PMC12484523; doi:10.1186/s41235-025-00671-0)
Supplement: Supplementary file 1 — Additional file 1. [file 41235_2025_671_MOESM1_ESM.docx]

**Supplementary Materials**

For “Using visual imagery to manipulate recognition memory for faces whose appearance has changed”

**Descriptive Statistics for Overall Memory Performance**

***Experiment 1***

The hit rate, defined as a recognition response of 5 or higher on old arrays, was .31 for congruent faces and .28 for incongruent faces. The false alarm rate, defined as a response of 5 or higher on new arrays, was .17 (note that the condition manipulation did not affect new arrays). Raw forced-choice accuracy (i.e., not subject-averaged) was .49 for congruent faces, and .49 for incongruent faces.

***Experiment 2***

The hit rate, defined as a recognition response of 5 or higher on old arrays, was .33 for congruent faces and .31 for incongruent faces. The false alarm rate, defined as a response of 5 or higher on new arrays, was .19 (note that the condition manipulation did not affect new arrays). Raw forced-choice accuracy (i.e., not subject-averaged) was .49 for congruent faces, and .48 for incongruent faces.

**Figure S1**

*Forced-choice Accuracy by Condition and Recognition Response*

*Note*. As in the figures in the main text, vividly imagined trials are included. A) Experiment 1. B) Experiment 2.


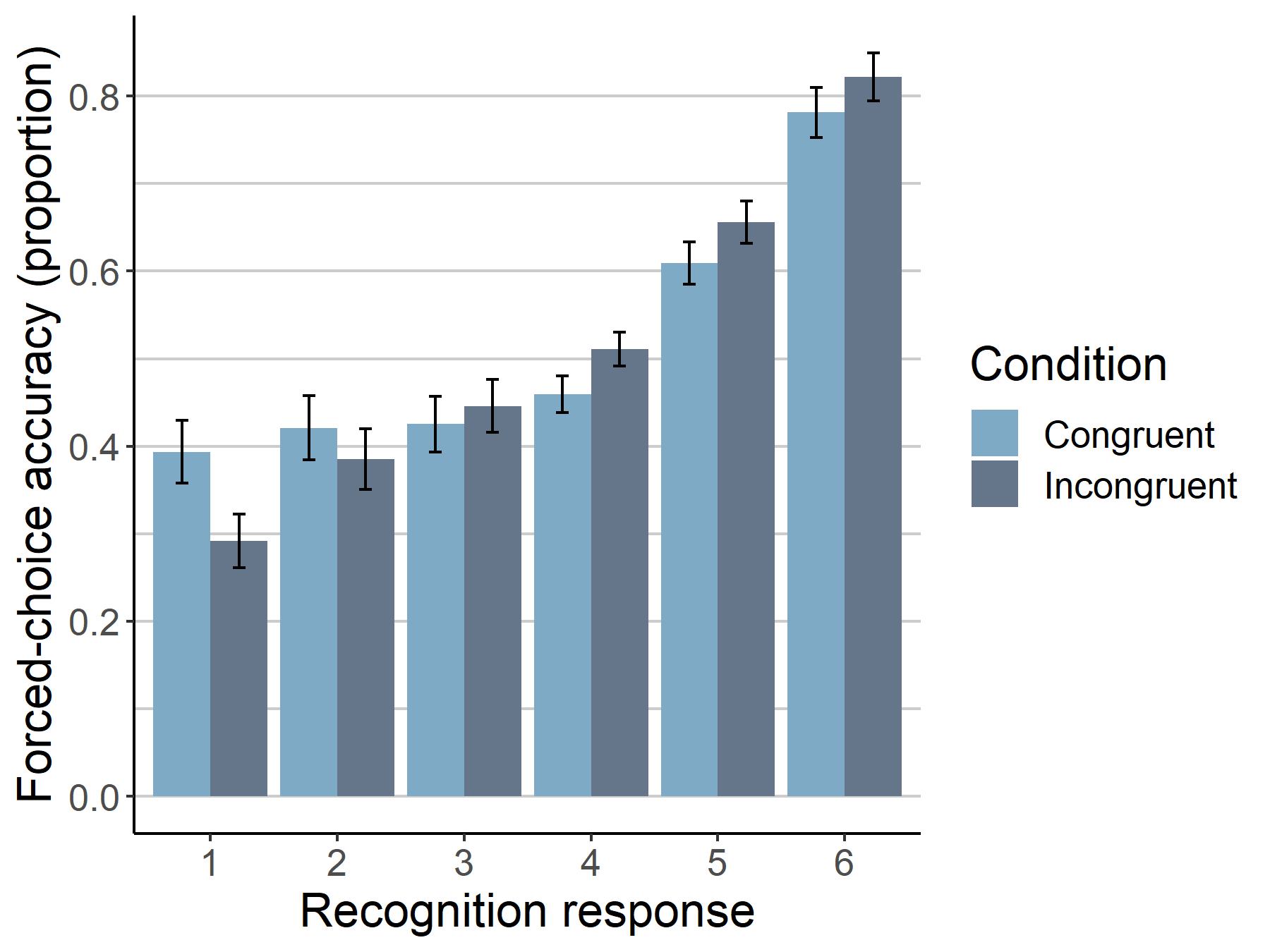

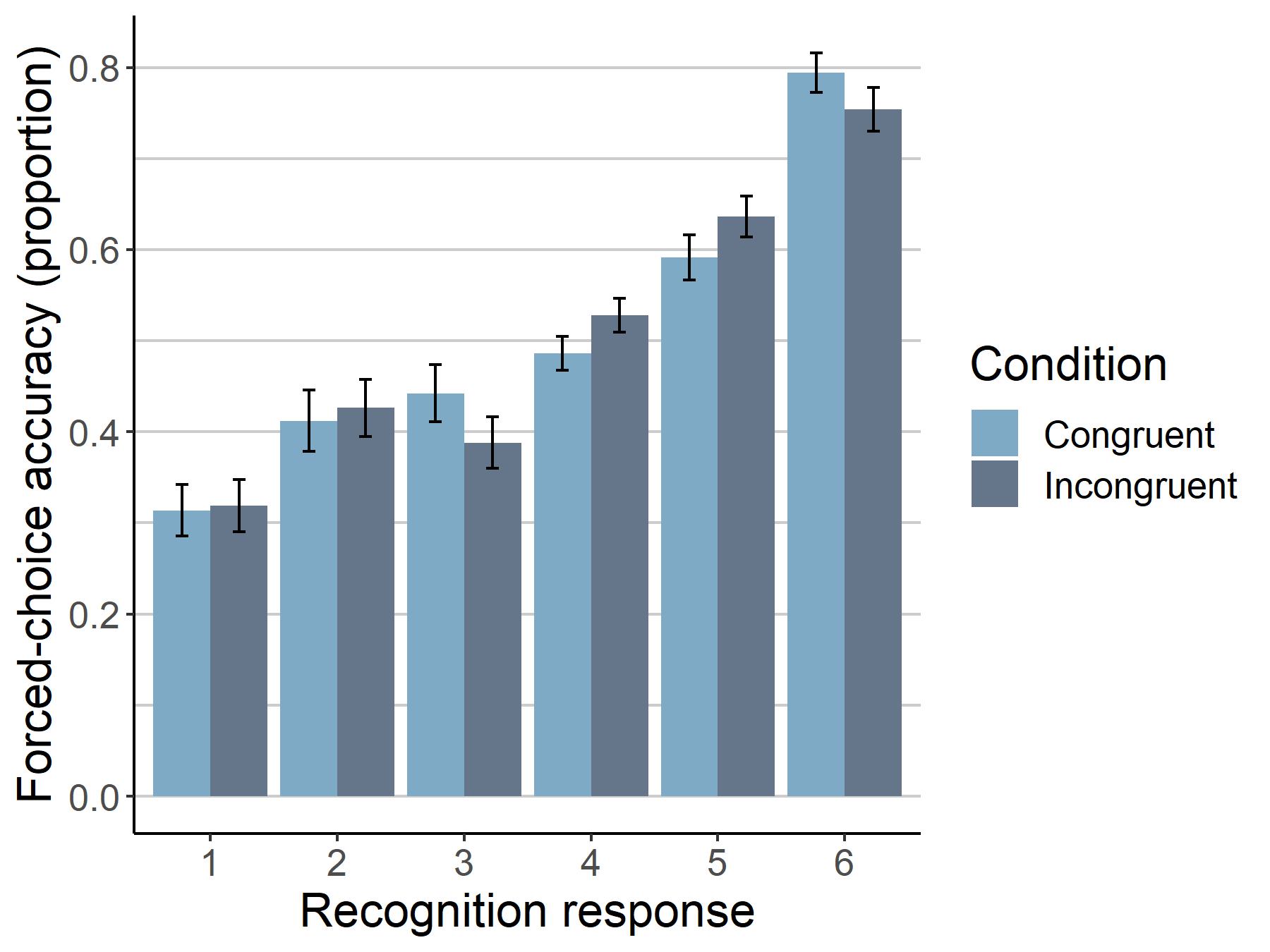


**A)**

**B)**

**Analyses by Facial Expression**

Given the importance of facial expression in recognition (e.g., D’Argembeau et al., 2003), we also examined whether our effects were moderated by expression (happy vs angry). In Experiment 1, there was no significant effect of facial expression on the size of the imagery congruency effect on AUC, *SMD*=-.14, *p*=.30. In Experiment 2, we found a significant effect of facial expression on the size of the imagery congruency effect on AUC, *SMD*=-.25, *p*=.04, with a larger effect for happy than angry faces.
